# Rheological properties of polyether polyurethane rubber based magnetorheological elastomers under transverse shear and vertical pressure

**DOI:** 10.1371/journal.pone.0312496

**Published:** 2024-11-21

**Authors:** RunPu Li, Fei Guo, Chengbin Du, Jiming Gu

**Affiliations:** 1 Nanjing Vocational Institute of Railway Technology, Nanjing, China; 2 School of Civil Engineering and Architecture, Anhui Polytechnic University, Wuhu, China; 3 Hohai University, Nanjing, China; 4 School of Management Science and Engineering, Anhui University of Technology, Ma’anshan, China; National University of Sciences and Technology, PAKISTAN

## Abstract

A novel magnetorheological vibration isolator with two operating conditions, horizontal shear and vertical compression, was designed and manufactured. The rheological properties of the energy-dissipating material were directly related to the volume fraction of iron powder in the laminated working unit of the magnetorheological vibration isolator. Aggregation of the carbonyl iron powder (CIP) strongly influenced on the rheological properties of the magnetorheological vibration isolator. Considered that the curing temperature affected the preparation process, polyurethane rubber was selected as the collective matrix of the magnetorheological elastomer (MRE) because of its wear resistance, good adhesion, high strength, corrosion resistance and solvent resistance. The dynamic properties of the polyurethane rubber MREs were experimentally characterised. A mathematical model was established for the magnetic induction effect (MIE) of the polyurethane magnetorheological isolator in a transverse shear deformation mode as well as a vertical tension and compression deformation mode. The magnetorheological effect was strongest under transverse shear deformation for an effective volume fraction of particles of 34% because of the effect of aggregation of the iron powder particles. The magnetic compression modulus depended strongly on the strain under vertical compression.

## 1. Introduction

Magnetorheological elastomers (MREs) are compound materials consisting of a nonmagnetic elastomer matrix and ferromagnetic particles [1]. The stiffness and damping characteristics of MREs change under an external magnetic field [2–4], and the magneto-induced effect (MIE) or magnetorheological effect (ME) of MREs has been widely exploited in industrial technology [5, 6]. A mechanical model of the MIE is required to optimize the design of an MRE vibration isolator. However, studies have only been preformed on classical viscoelastic and macro-mechanical models based on the MRE force–displacement relationship and micromechanical models of the MRE at the particle scale [7], such as the five elements [8] and particle chain model [9–11]. In fact, the distribution of ferromagnetic particles is uneven or irregular in the nonmagnetic elastomer matrix, resulting void defects and agglomerations of particles [12]. The particle arrangement in an MRE has a major effect on the macroscopic behaviour of these smart materials [13, 14]. The influence of nonideal interfaces on the viscoelastic behaviour of an MRE was analysed using the Mori–Tanaka model [15], in which the MIE of MR materials is added to the constitutive relationship of composite materials, but without considering the effective volume fraction of the particles. Most research results have been obtained using mechanical models of materials.

The content of ferromagnetic particles in an MRE strongly affects the magnitude of the MIE [16]; as a result, MREs have not been commercialized except as shock absorbers and for vibration control [17, 18]. A shear model of an MRE with a single degree of freedom was studied developed for a bidirectional shear MRE vibration isolator [19]. Winger et al. [20] reported that the microparticle fraction plays a critical role in the magnetorheological (MR) effect. Considering the actual working state of an MRE, Vatandoost [21] pre-strained an MRE specimen and determinded how the particle volume fraction and frequency affected the stress‒strain relationship. Afiq Azri Zainudin [22] used cobalt particles with magnetic-electrical properties as fillers in an MRE and found that the rheological and electrical properties of the MRE improved as the cobalt content of the silicone matrix was increased. The interface between magnetic particles and elastomers is a critical influence factor of the MR effect of MREs. Jiaqing Zhao [23] used polydopamine (PDA) deposition and n-dodecyltrimethoxysilane (DTMS) addition to improve the interfacial interaction between rubber and carbonyl iron (CI) particles and thereby enhance the mechanical properties of MREs.

Stiffness and damping must be carefully balanced to optimize the vibration isolation performance of a system. An excessively large stiffness can result in a harsh response to high-frequency disturbances, whereas insufficient damping may allow excessive movement and long settling times. Lin Chen [24] used parallel or series combinations of an NSD and a viscoelastic damper to improve the performance of dampers on a stay cable. Dezhao Lin [25] proposed a novel vibration isolator that integrates tuneable stiffness–damping and active driving properties by radially embedding iron chains into a magnetorheological elastomer (MRE). The inherent adjustable stiffness–damping and active driving force produced by the MRE material can be harnessed to construct a fail-safe MRE vibration isolator. In this study, a novel magneto-viscoelastic model was formulated for both isotropic and anisotropic MREs considering the effects of preparation, and the magnetic field intensity during rheometric testing [26].We focus on the influence of the particle volume fraction on the magnetorheological effect of a novel MR isolation and damping support, which consisted of an MR plastic working unit and an MRE working unit [27, 28].

## 2. Preparation of a polyether polyurethane MRE

Generally, MREs consist of a magnetizable solid phase dispersed in a nonmagnetic carrier such as an elastomer. CIP is used as the magnetizable solid phase. There are many possible matrix rubber materials, such as natural rubber [29], silicone rubber [30, 31], butyl rubber, chlorosulfonated polyethylene rubber, nitrile rubber, neoprene rubber, and acrylic rubber [32]. Butyl rubber has poor processing and bonding performance [33]. The directionality of magnetic particles in soft MR materials strongly affect the MRE mechanical properties along the magnetic field direction [34], and ferromagnetic particles with irregular shapes and large equivalent radii can increase the MIE [35].

The new rubber with an unsaturated structure has good elasticity and toughness. Rubber can be vulcanised using either peroxide or sulfur. New rubber is ideal for preparing high-performance MREs. In this study, polyether polyurethane rubber (PPR) was used as the MRE matrix. The raw ferromagnetic particles were made of CIP. Jiangsu Tianyi Metal Powder Co., Ltd. produced the iron powder containing mainly spherical particles with an average size of 3 μm. The main performance index parameters of the iron powder are shown in Tables 1 and 2. Zinc oxide, magnesium oxide, stearic acid, and N-330 carbon black were used as additives, 2,2’-dithiodibenzothiazole (DM) was used as an accelerator, Dioctyl phthalate (DOP) and methyl silicone oil were used as plasticizers, and IS-60 sulfur and dicumyl peroxide (DCP) were used as vulcanising agents.

**Table 1:** The performance index of the CIP (mass composition shown).

Fe	C	N	O	Size	Density
98.03%	0.74%	0.86%	0.36%	3.0μm	2.8–4.25 g·cm^-3^

**Table 2:** Particle size distribution (μm).

D10	D50	D90
<1.8	<3.5	<8.0

**Fig 1:**
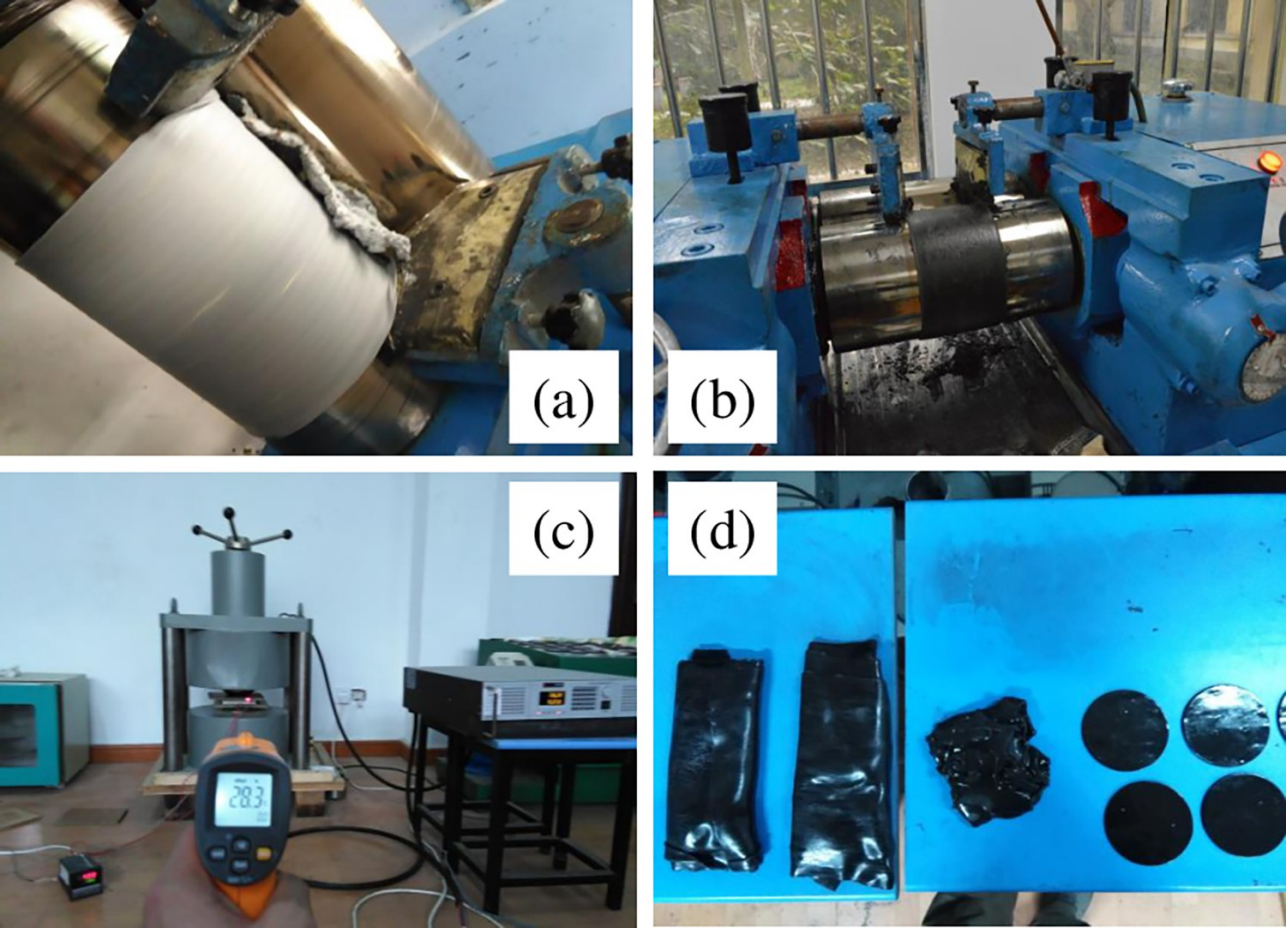
The MRE preparation process. (a) matrix plasticization, (b) component mixing, (c) prestructuring, and (d) curing.

**Fig 2:**
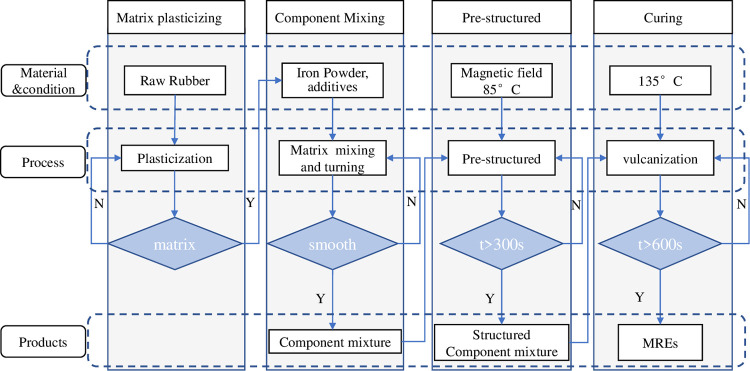
Fabrication process of MR materials.

**Fig 3:**
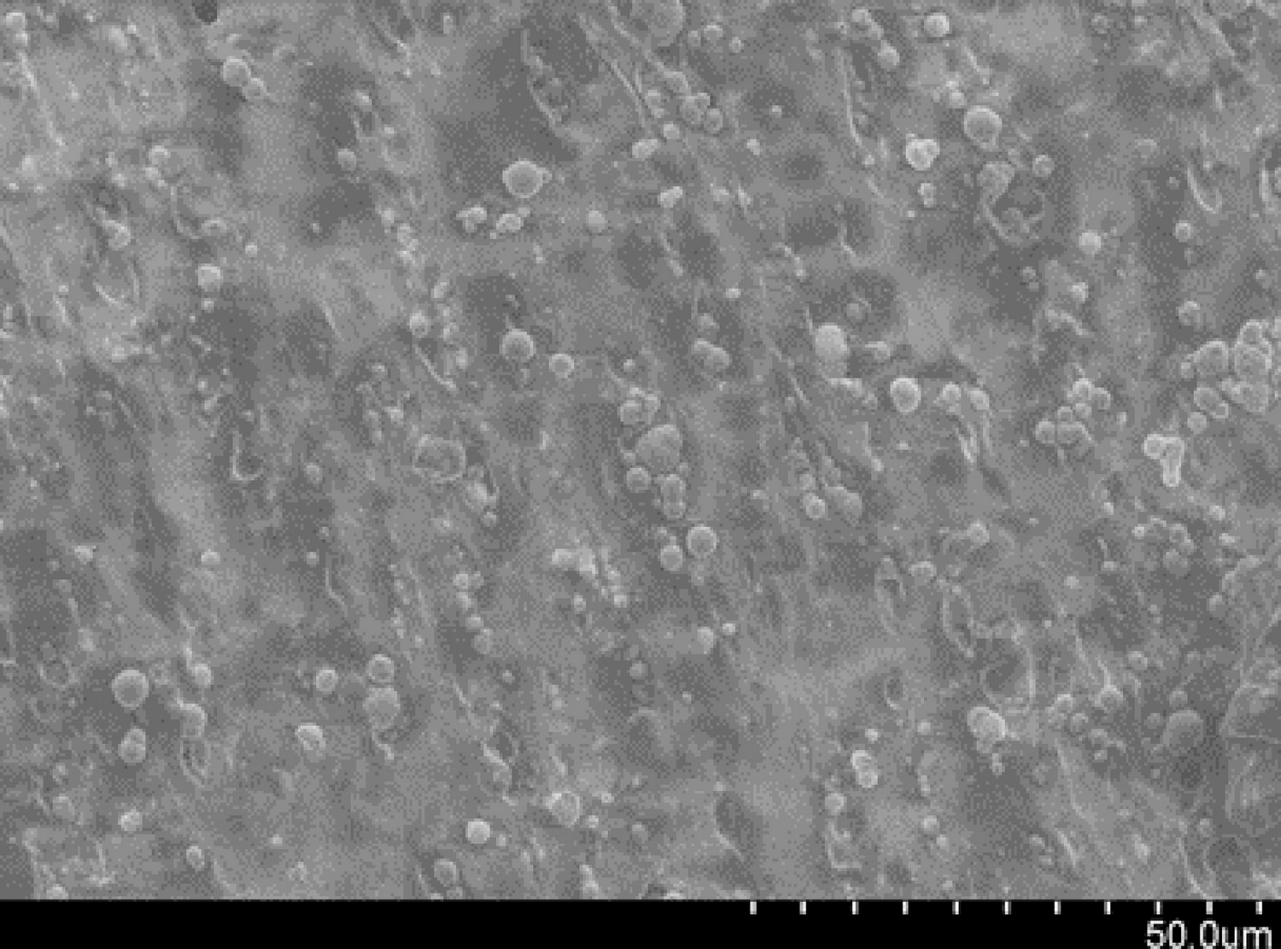
Microcharacterisation of the MREs.

Fig 1 shows the MRE preparation process of four main steps: matrix plasticization, component mixing, pre-structuring, and curing. In the first step, the macromolecular chains of the raw rubber were broken by a shear force into active free radicals, which then combined with other free radical acceptors under the action of oxygen and heat. Second, the ferromagnetic particles, accelerators, active agents, antioxidants, carbon black and other additives were blended into a mixed plastomer. Prestructuring was carried out in an aluminium mould at a magnetic induction intensity of 1.2 T and an aluminium plate temperature of 85°C. The curing process consisted of vulcanization at 135°C, during which the linear rubber molecules were chemically cross-linked into a three-dimensional network, as shown in Fig 2. Fig 3 shows an SEM image obtained using a Hitachi S4800 scanning electron microscope, illustrating an aggregate-chain dispersion of CIP in the matrix, which is crucial for the performance of the MREs under magnetic fields. The MRE components are shown in Table 3.

**Table 3:** MRE component.

Carbonyl Iron powder	Polyurethane raw adhesive	plasticizer	carbon black	Others
1349	100	100	20	18

## 3. Testing and analysis of the MREs

The shear storage modulus reflects the storage and release of deformation energy in an MRE during one rotation as well as the elastic properties. The loss factor represents the ratio of the energy consumed to the maximum strain energy of the MRE during the vibration period and is an index of its energy dissipation and damping ability of the MRE. The rotational shear performance of an MRE is assessed in terms of the shear storage modulus and loss factor. Johari [36] used a magnetorheometer (MCR 302) to systematically investigate how the weight percentage (wt%) and arrangement of particles affected the MRE storage modulus. Gan [37] comprehensively studied the effect of the particle size on the viscoelastic properties of MREs by using an advanced rheometer (Model: MCR301, Anton Paar) to examine the influences of the frequency, strain, and magnetic field on samples with different particle sizes. The rheological properties of the different samples were measured with a rheometer (Physica MCR 302) from Anton Paar Co., Graz, Austria [38].

**Fig 4:**
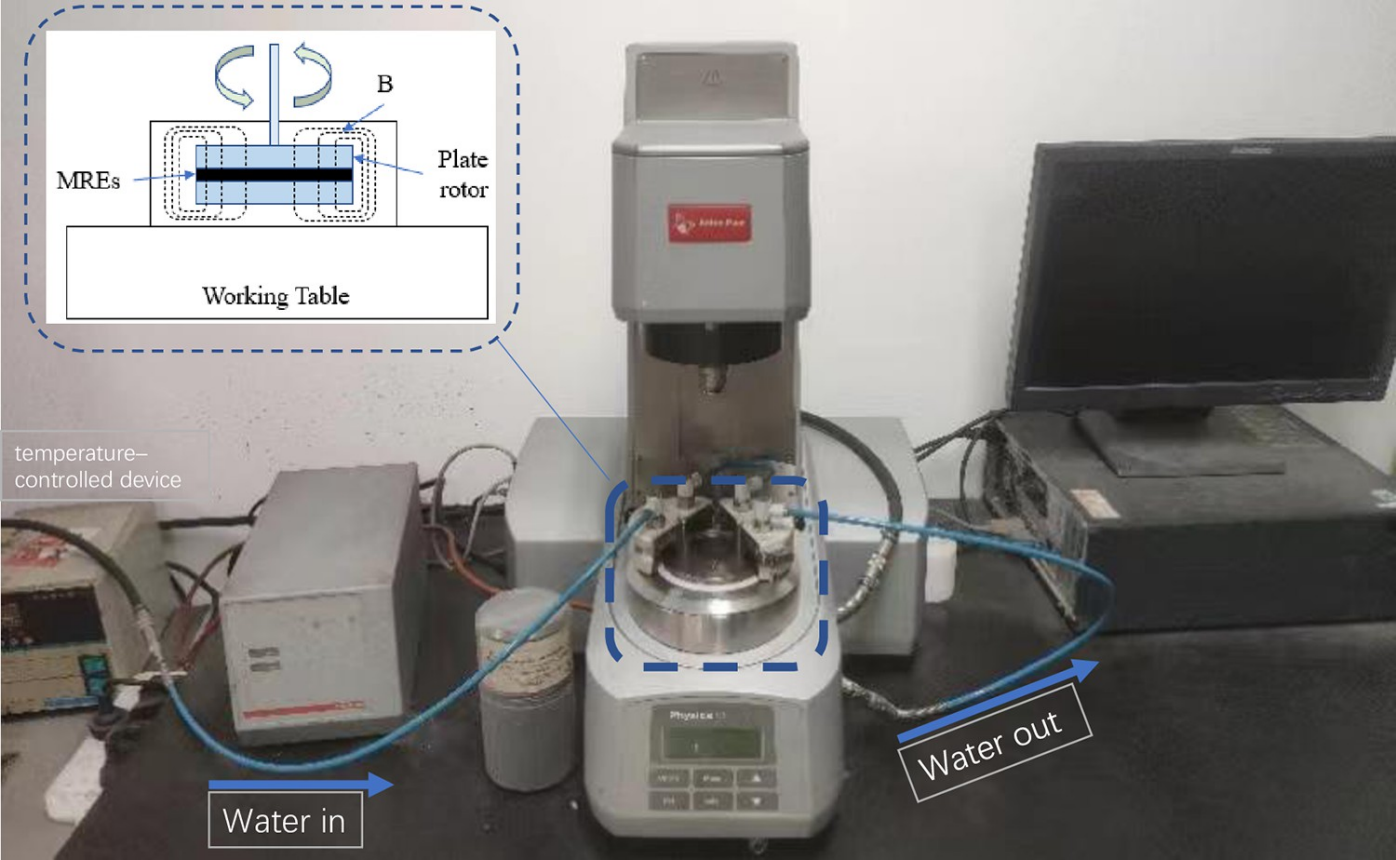
The MCR51 rheometer.

The MRE was laid uniformly between the two parallel disks of an MCR51 rheometer, as shown in Fig 4, and a sinusoidal excitation force (shear frequency: 1 Hz; strain amplitude: 0.1%, temperature: 30°C) was applied to measure the storage modulus and loss factor of the material.

**Fig 5:**
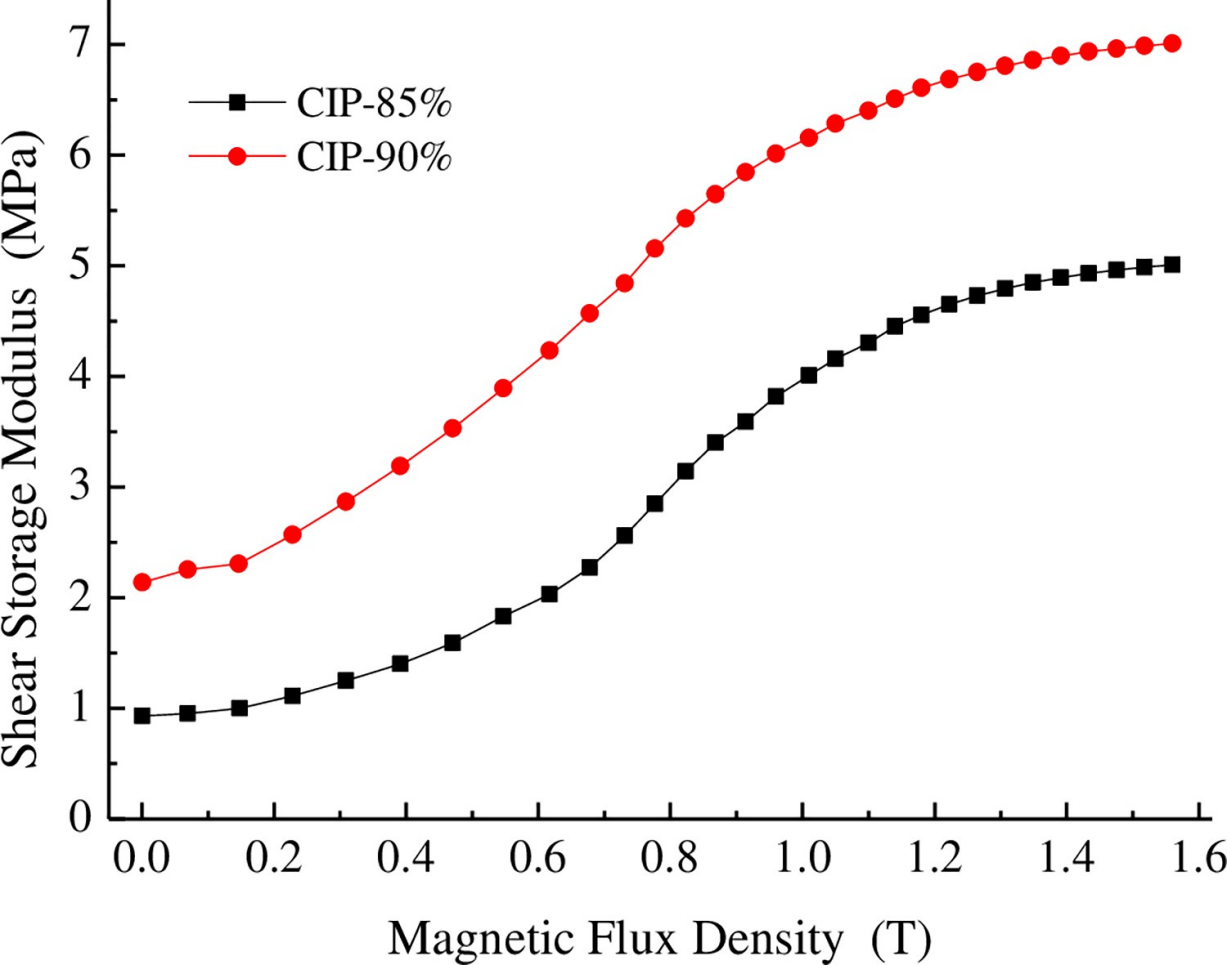
Dependence of the storage modulus on the magnetic flux density.

**Fig 6:**
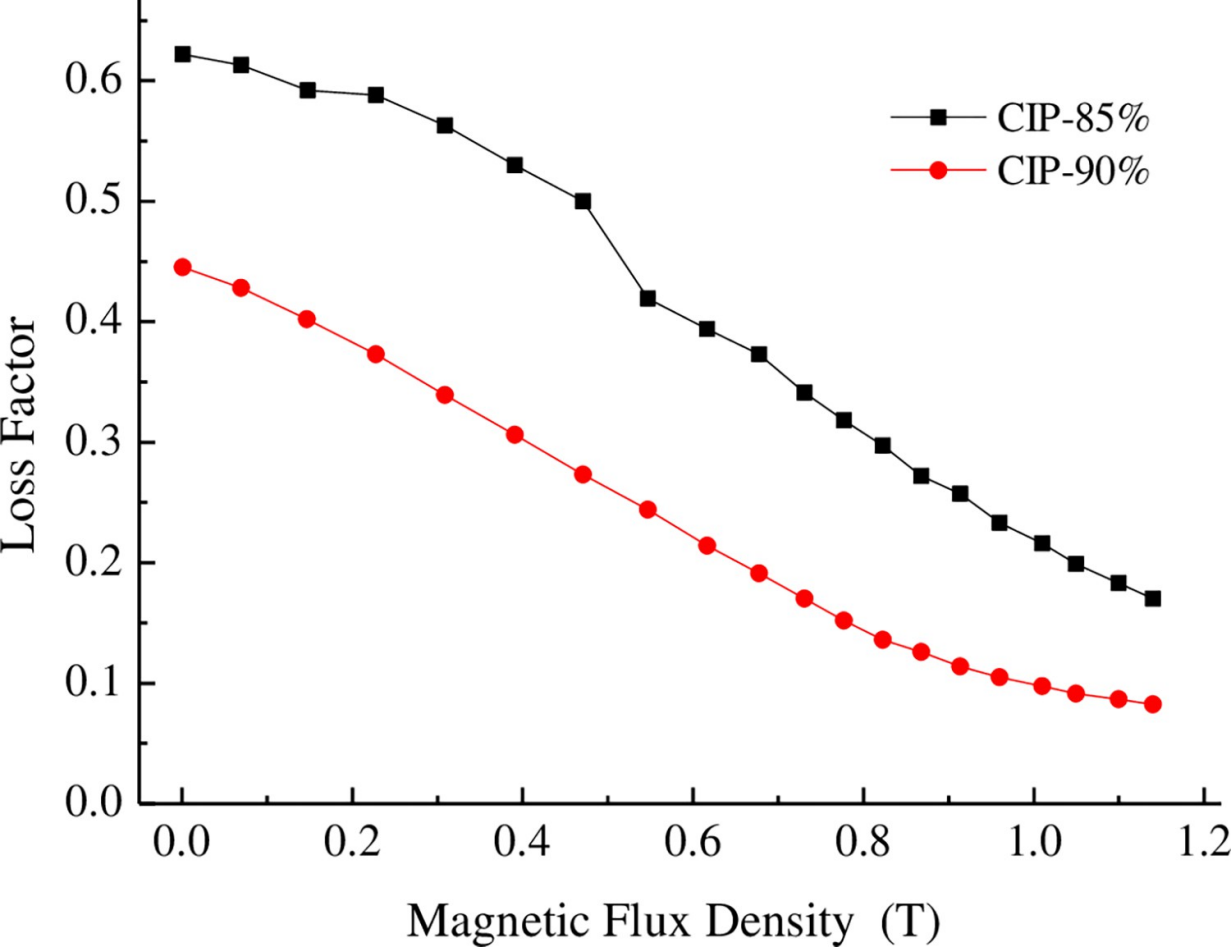
Dependence of the loss factor on the magnetic flux density.

The influence of CIP content on the storage moduli of the MRE is shown in Fig 5. Fig 6 shows that adding 90% CIP to the rubber-based MRE changed the absolute modulus by 5 MPa and the relative modulus by 228% compared to the MRE with 85% CIP. The increase in the iron powder content led to a tighter internal particle structure of the MRE, as the particles were more compactly arranged, and increased the initial modulus. The relative modulus of the MRE decreased sharply as the CIP content increased. The larger the content of CIP was, the smaller the loss factor was.

## 4. Design and preparation of an MRE isolation support

**Fig 7:**
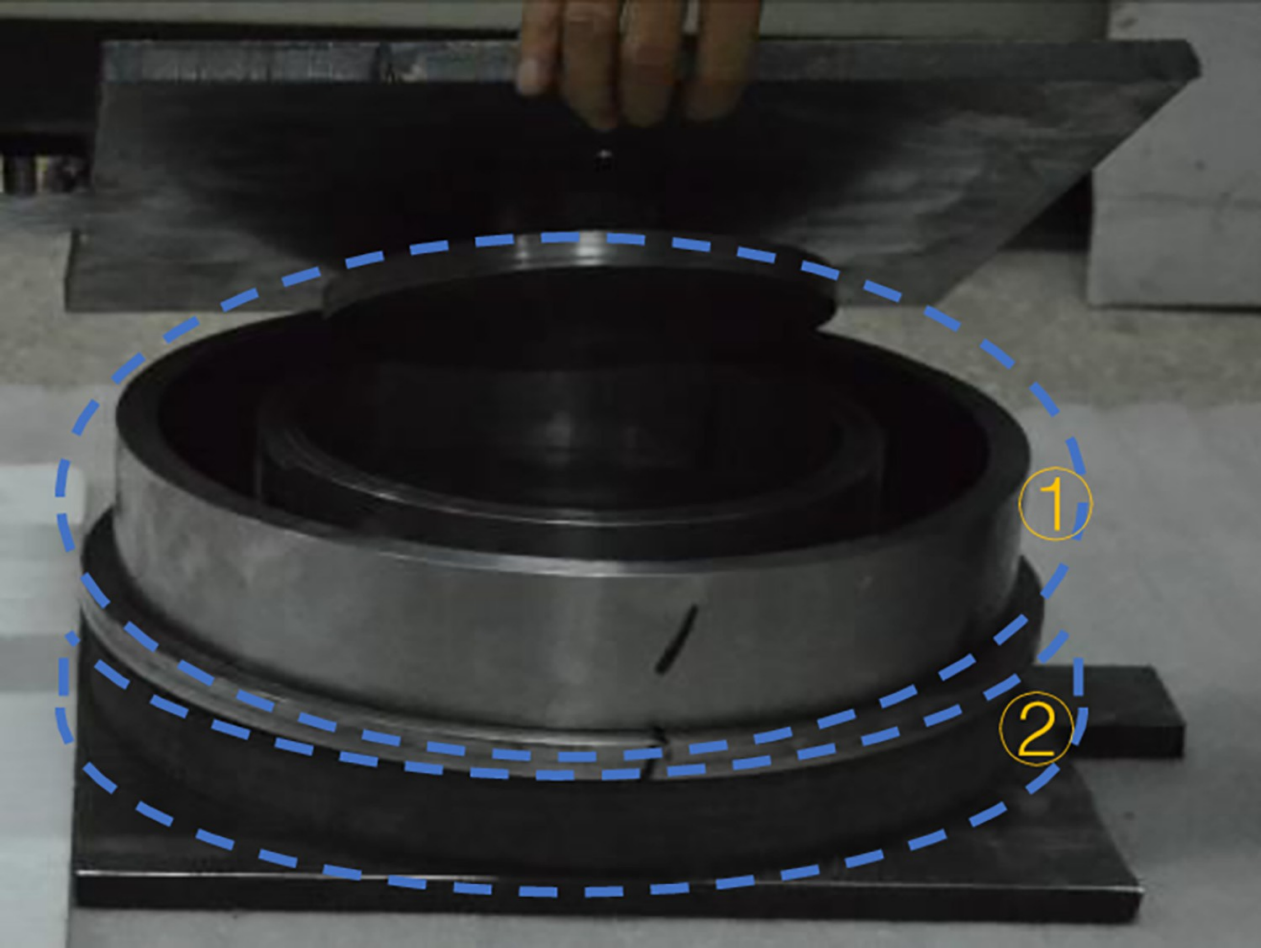
The MRE isolation support.

**Fig 8:**
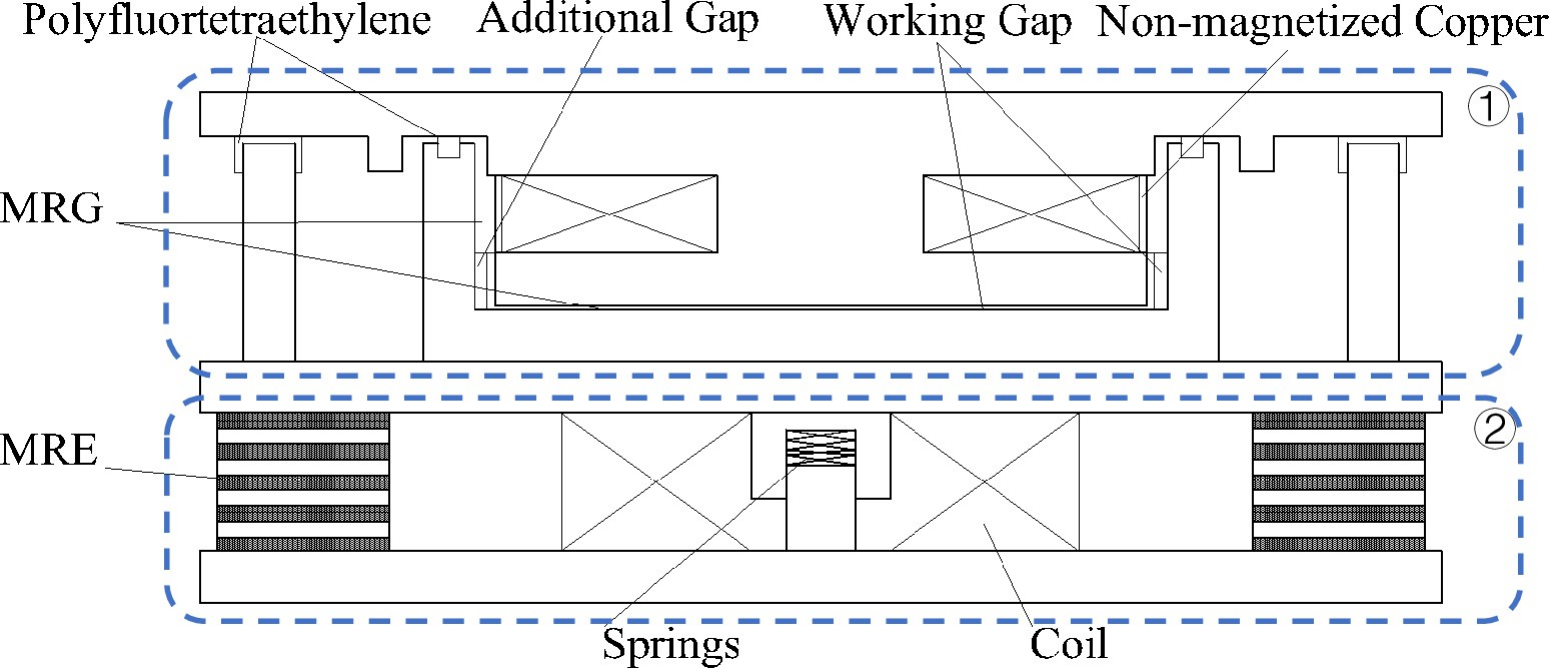
Diagram of the novel MR seismic isolation bearing.

An MRE isolation support [27] was designed and prepared as shown in Fig 7. This novel MR support for isolation and damping consisted of two working units, an MR plastic body (①, upper part) and an MR elastomer (②, under part). Stiffness and damping were provided by the MR plastic body and the MRE under small and large displacements, respectively. The damping material of the lower working unit of the MRE was a PPR matrix as shown in Fig 8. The main design parameters of the MR seismic isolation bearing are shown in Table 4.

**Table 4:** Design parameters of the MR seismic isolation bearing.

Outer diameter of the laminated body	350 mm
Inner diameter of the laminated body	200 mm
Steel plate thickness	2 mm
Travel limit of the working disc	50 mm
Number of turns of the working disc coil	1600
Total height	150 mm

## 5. MIE under transverse shear deformation

A prescribed vertical pressure was applied to the MRE in the lower working unit of the novel MR vibration isolation support during operation. Each ferromagnetic particle was assumed to be a magnetic dipole [9]:

$$\vec{m_{i}}=\frac{4}{3}\pi r^{3}\mu_{0}\mu_{1}\chi H_{i}$$ (1)

where *μ*_0_ = 4π×10^7^ *H*/*m* is the vacuum permeability, *μ*_1_is the relative permeability of the polyether polyurethane rubber-based MRE, and *X* is the particle magnetic susceptibility.

The interaction energy *E*_12_ of two adjacent dipoles $\vec{{,m}_{i}}$ and $\vec{m_{j}}$, can be expressed according to magnetic dipole theory as follows:

$$E_{12}=\frac{\vec{m_{i}}\vec{\cdot m_{j}}-3(\vec{m_{i}}\cdot \vec{d})(\vec{m_{J}}\cdot \vec{d})∕d^{2}}{4\pi \mu_{0}\mu_{1}d^{3}}$$ (2)

where *d* is the distance between the centres of the two neighbouring magnetic particles.

Then, the magnetic-field intensity *H*_*i*_ in the locality of aggregated particle *i* can be expressed as:

$$H_{i}=\vec{H_{0}}+\sum_{j\neq i}H_{j}=\vec{H_{0}}+2\sum_{j=1}^{n}\frac{3\hat{d_{j}}(\hat{d_{j}}∙\vec{m_{j}})-\vec{m_{j}}}{4\pi \mu_{0}\mu_{1}{\left(d_{j}\right)}^{3}}$$ (3)

where $\vec{H_{0}}$ is the intensity of the external magnetic field and $\hat{d_{j}}$ is the unit vector of $\vec{d_{j}}$. Then,

$$\vec{m_{i}}=\frac{4}{3}\pi r^{3}\mu_{0}\mu_{1}\chi \left[\vec{H_{0}}+2\sum_{j=1}^{n}\frac{3\hat{d_{j}}(\hat{d_{j}}∙\vec{m_{j}})-\vec{m_{j}}}{4\pi \mu_{0}\mu_{1}{\left(d_{j}\right)}^{3}}\right]$$ (4)

Disregarding the influence of the dynamic Poisson’s ratio and the bending deformation of the aggregate chain results in the simpler one-dimensional shear problem *D* = *D*_0_(1−*ε*_*matrix*_) where *D*_0_ is the distance between adjacent aggregates at σ = 0 and *ε*_*matrix*_ = *σ*/*E*_*matrix*_ is the strain in the polyether polyurethane rubber matrix. As part of the preparation process, the polyether-polyurethane-rubber-based MRE was prestructured in an external magnetic field. As the aggregates of the resulting magnetized particles were more rigid than the matrix, the deformation of the MRE was considered to be matrix deformation. For *m*_*i*_ = *m*_*j*_ = *m*,

$$\vec{m_{i}}=\frac{4}{3}\pi r^{3}\mu_{0}\mu_{1}\chi \left[\frac{3k_{0}^{3}{\left(1-\varepsilon_{matrix}\right)}^{3}}{3k_{0}^{3}{\left(1-\varepsilon_{matrix}\right)}^{3}-4\chi A}\right]$$ (5)

where *r* is the average aggregate radius and *k*_0_ is the ratio of *D*_0_ to *D*. At $\sigma =0,A=\sum_{j=1}^{n}\frac{1}{j^{3}}$; at a sufficiently large n, *A* = 1.202. The magneto-induced shear modulus of the MRE is:

$$\Delta G=\frac{9}{8}\frac{\varphi A(4-\gamma^{2})m^{2}}{d^{3}\pi^{2}r^{3}\mu_{0}\mu_{1}{\left(1+\gamma^{2}\right)}^{7/2}}=\frac{18\mu_{0}\mu_{1}^{m}\chi^{2}H^{2}\varphi Ak_{0}^{3}{\left(1-\varepsilon_{0}\right)}^{3}(4-\gamma^{2})}{{\left(1+\gamma^{2}\right)}^{7/2}{\left[3k_{0}^{3}{\left(1-\varepsilon_{0}\right)}^{3}-4\chi A\right]}^{2}}$$ (6)

The MR material of the vibration isolation support operates in the elastic regime, and the material properties of the lower working unit of the MRE are determined at the onset of shear deformation. The shear magneto-induced shear modulus of the MRE at small shear deformation displacements can be expressed as

$$\Delta G=\frac{9}{2}\frac{\varphi Am^{2}}{d^{3}\pi^{2}r^{3}\mu_{0}\mu_{1}}=\frac{72\mu_{0}\mu_{1}^{m}\chi^{2}H^{2}\varphi Ak_{0}^{3}}{{\left[3k_{0}^{3}-4\chi A\right]}^{2}}$$ (7)

The magnetic shear modulus of the MRE should be defined as a function of the particle volume fraction. Although the particle mass fraction can be measured directly, the particle volume fraction can be more conveniently used in calculations, and is given by $\frac{\rho_{\mathrm{CIP}}}{\rho_{\mathrm{matrix}}}(\frac{1}{\varphi_{m}}-1)=\frac{1}{\varphi_{v}}-1$, where *ρ*_CIP_ and *ρ*_matrix_ are the densities of the CIP and matrix, respectively). The particle volume fraction can be written as

$$\varphi =\frac{\sum_{i=1}^{n}\frac{4}{3}\pi r^{3}}{\sum_{i=1}^{n}\frac{4}{3}\pi d_{0}^{3}+V_{edge}}$$ (8)

where *V*_*edge*_ is the matrix volume. Formula (6) can be rewritten using an adjustment coefficient *a k*_0_ = (*aφ*)^−1/3^ as

$$\Delta G(\varphi )=\frac{72Aa\mu_{0}\mu_{1}^{m}\chi^{2}H^{2}\varphi^{2}}{{\left[3a\varphi -4\chi A\right]}^{2}}$$ (9)

**Fig 9:**
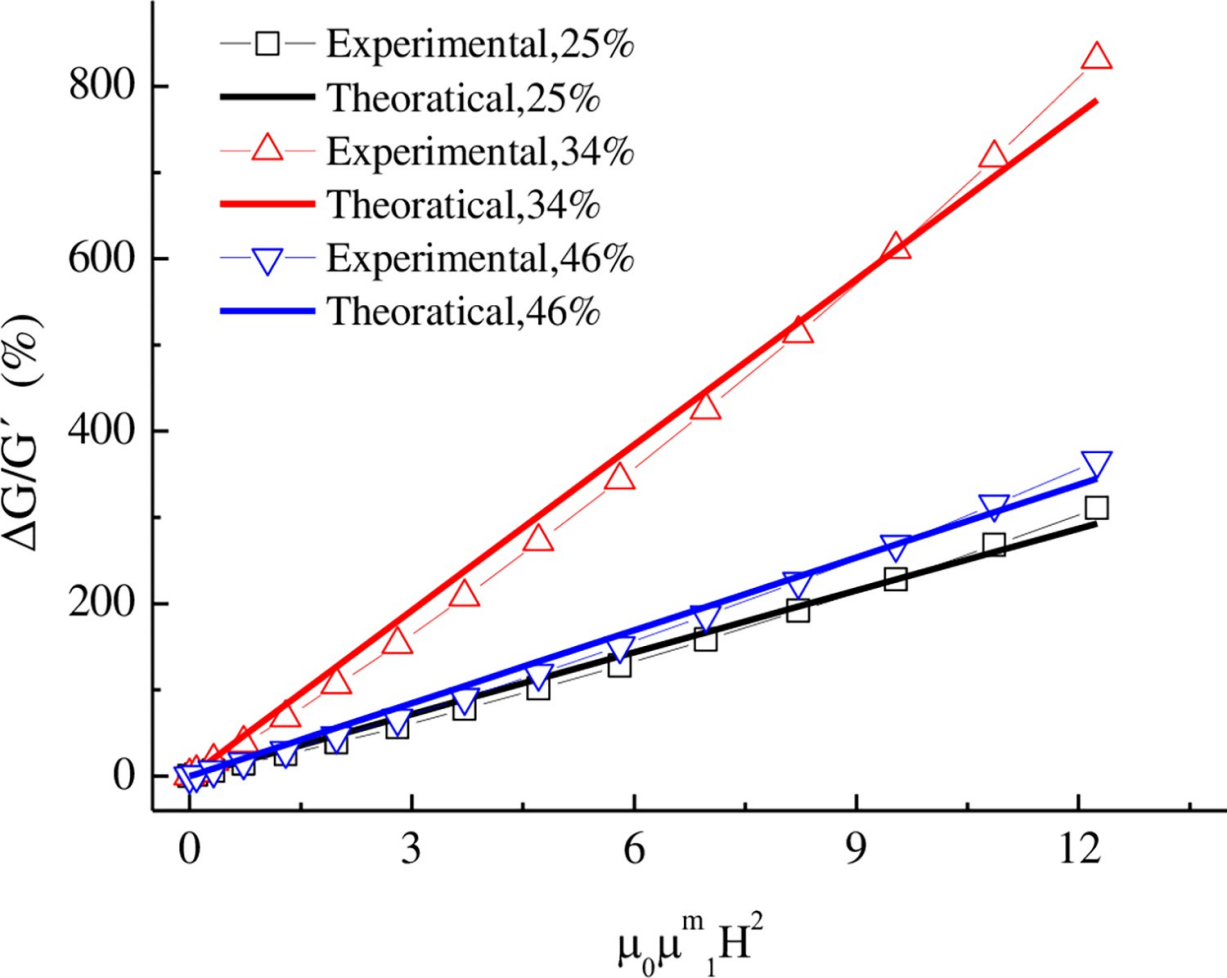
Dependence of the MR effect on the dimensionless field strength under transverse shear deformation.

The relationship between the MIE of the MRE and the magnetic field strength is shown in Fig 9. Under transverse shear deformation, the magneto-induced effect of the MRE increases with both the particle volume fraction and the magnetic field strength. The MIE for the MRE with a particle volume fraction of 46% was larger than that for the MRE with a particle volume fraction of 25% but smaller than that for the MRE with a particle volume fraction of 34%. This result was obtained because the initial shear modulus Δ*G*′ increased with the particle volume fraction. The adjustment coefficient *a* was 5.088, 2.649 and 2.907 at particle volume fractions (φ) of 25%, 34%, and 46%, respectively.

## 6. MIE under vertical tension and compression deformation

When vibration isolation supports are installed at a site, the lower working units are compressed. Therefore, the influence of the vertical strain on the magneto-induced effect under vertical deformation was investigated. The classic Jolly mechanical model is widely used to describe interactions between particles in infinite particle chains and interactions between particle chains [38]. However, using this theory results in a negative MRE modulus regardless of whether the intensity of the external magnetic field is increasing or decreasing, the modulus of the MRE calculated according to this theory was negative [39]. In this study, energy theory was used to determine the influence of the volume fraction of ferromagnetic particles on the magneto-induced effect.

**Fig 10:**
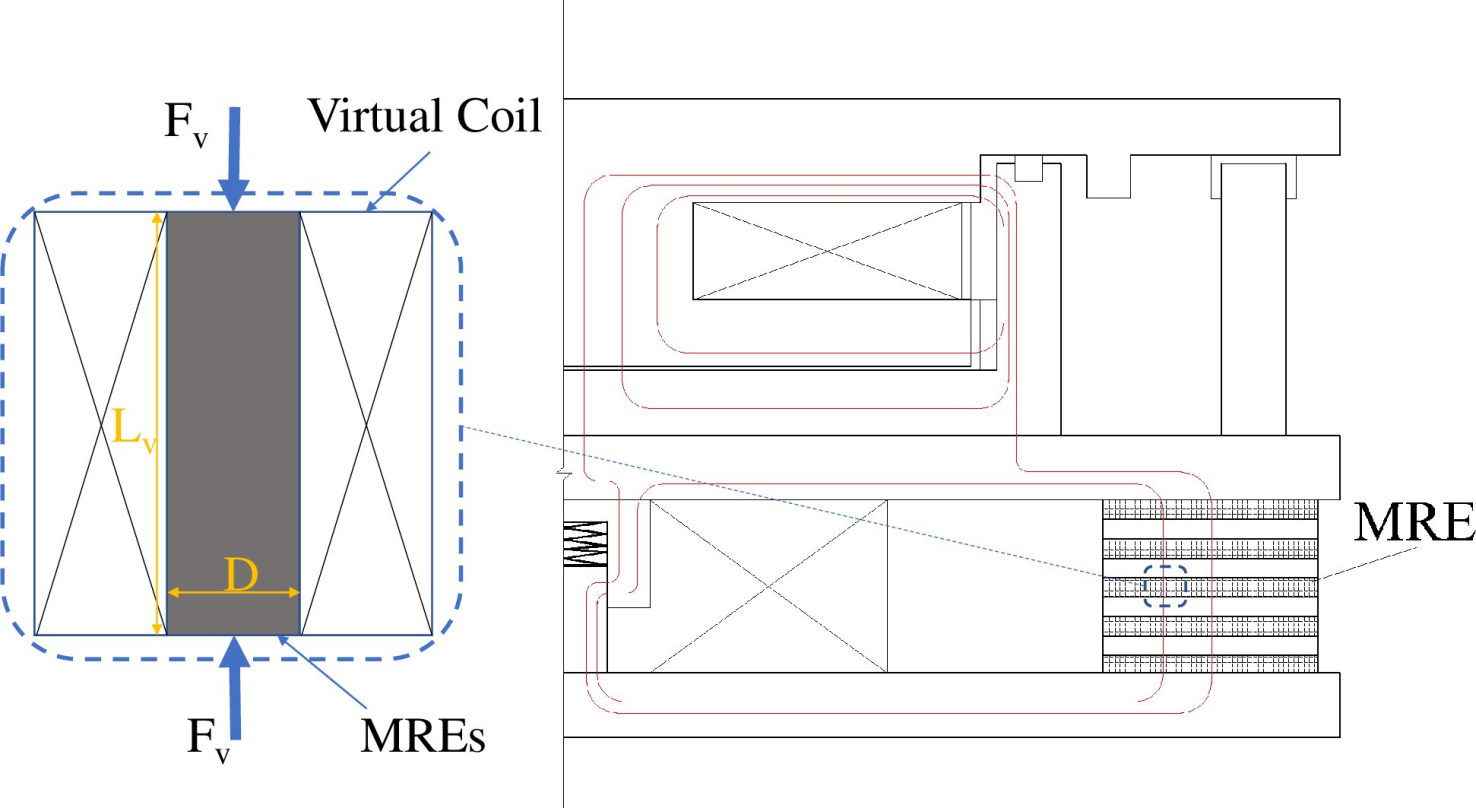
Unit cylinder and its virtual magnetic field.

The external magnetic field was applied parallel to the orientation of the particle chain in the MRE lower working unit. A control volume of a unit cylinder with a diameter *D* and height *L*_*v*_ in the MRE was considered, and a unit vertical force *F*_*v*_. was applied to this cylinder. A magnetic field *H* passing through the unit cylinder was modelled as a virtual electromagnetic coil with a wire that could expand and contract freely without changing the cylinder deformation, as shown in Fig 10.

In the system of the unit cylinder and magnetic field, the free energy was assumed to be reversible and temperature changes were neglected. The Helmholtz free energy theory was used to calculate the change in the free energy of the cylinder under a small vertical deformation as

$$\Delta E=\Delta F_{v}l_{v}+\Delta F_{r}l_{r}+NI\Delta \emptyset$$ (10)

where *l*_*v*_ is the vertical deformation, *F*_*r*_ is the unit radial force, *l*_*r*_ is the radial deformation, *N* is the number of turns of the equivalent electromagnetic coil along the cylinder perimeter, *I* is the intensity of the virtual current in the coil, and ∅ is the magnetic flux through the cylinder cross-section. Then, the free energy per unit volume of the MRE is

$$dE=\frac{4F_{v}}{\pi D^{2}}d\delta_{v}+\frac{2F_{r}}{\pi DL}d\delta_{r}+HdB$$ (11)

where *δ*_*v*_ and *δ*_*r*_ are the vertical and radial deformations of the MRE per unit volume, respectively, *δ*_*v*_ = *l*_*v*_/*L* and *δ*_*r*_ = 2*l*_*r*_/*D ε*_*v*_ and *ε*_*r*_ are the vertical and radial strains in the MRE per unit volume, respectively; then,

$$\varepsilon_{v}=\left\{ \begin{array}{c} 1-\delta_{v},compress\\ \delta_{v}-1,stretch \end{array}\right.,$$

$$\varepsilon_{r}=\left\{ \begin{array}{c} 1-\delta_{r},compress\\ \delta_{r}-1,stretch \end{array}\right.,$$ (12)

where *H* = *NI*/*L* is the strength of the magnetic field in the equivalent electromagnetic coil, and *B* = 4∅/(*πD*^2^) is the magnetic induction intensity of the unit cylinder.

The calculation was simplified by neglecting the volume change per unit volume of the MRE during vertical deformation, such that $\pi {l_{r}}^{2}l_{v}=\pi D^{2}L/4$, and

$$\left\{ \begin{array}{c} {\delta_{v}^{2}\delta }_{v}=1\\ 2\delta_{r}\delta_{v}d\delta_{r}+\delta_{r}^{2}d\delta_{v}=0 \end{array}\right.$$ (13)

As our objective was to analyse the magneto-induced effect of the MRE under vertical deformation, the free energy per unit volume of the MRE was considered as a function of the vertical deformation and magnetic induction intensity.


$$\frac{dE(\delta_{v},B)}{d\delta_{v}}=\frac{4F_{v}}{\pi D^{2}}-\frac{\delta_{r}}{\delta_{v}}\frac{F_{r}}{\pi DL}$$ (14)

$$\frac{dE(\delta_{v},B)}{dB}=H$$ (15)

The cross-sectional area and vertical length of the MRE unit cylinder unit body change under the vertical deformation. The magnetic energy density of the deformed unit cylinder was $W=\frac{\delta_{v}^{2}B_{0}^{2}}{2\mu }$. The change in the free energy of the cylinder caused by deformation consisted of the elastic deformation energy and the work done by the magnetic field force between ferromagnetic particles. Thus, Formula (11) for the unit cylinder can be written as

$$\frac{dE(\delta_{v},B)}{d\delta_{v}}=\frac{\partial (E_{e}(\delta_{v})+\frac{\delta_{v}^{2}B_{0}^{2}}{2\mu })}{\partial \delta_{v}}=\frac{\partial E_{e}(\delta_{v})}{\partial \delta_{v}}+\frac{\delta_{v}B_{0}}{\mu }-\frac{\delta_{v}^{2}B_{0}^{2}}{2\mu }\frac{\partial \mu }{\partial \delta_{v}}$$ (16)

$$\delta_{v}\frac{\partial E_{e}(\delta_{v})}{\partial \delta_{v}}+\mu H^{2}-\frac{\delta_{v}H^{2}}{2}\frac{\partial \mu }{\partial \delta_{v}}=\sigma_{v}-\sigma_{r}$$ (17)

where $\sigma_{v}=\frac{4F_{v}}{\pi l_{r}^{2}}$ is the vertical stress and $\sigma_{r}=\frac{F_{r}}{2\pi l_{r}l_{v}}$ is the radial stress on the deformed cylinder. The normal stress on the plane perpendicular to the magnetic field direction is $\frac{\mu^{2}H^{2}}{2\mu_{0}}$, and the normal stress on the plane parallel to the magnetic field direction is $\frac{1}{2}\mu_{0}H^{2}$. The microscopic model used was transversely isotropic, and the external magnetic field was uniform along the direction of the particle chain; thus, the radial stress *σ*_*r*_ on the deformed cylinder after deformation was constant. Then, the tensile compressive modulus of the MRE could be expressed as

$${E_{v}(\varepsilon }_{v})=\frac{\partial \sigma_{v}}{\partial \varepsilon_{v}}=\left\{ \begin{array}{c} (1-\varepsilon_{v})\frac{\partial^{2}E_{e}(\varepsilon_{v})}{{\partial \varepsilon_{v}}^{2}}-H^{2}\frac{\partial \mu }{\partial \varepsilon_{v}}-\frac{1}{2}(1-\varepsilon_{v})H^{2}\frac{\partial^{2}\mu }{{\partial \varepsilon_{v}}^{2}},compress\\ (1+\varepsilon_{v})\frac{\partial^{2}E_{e}(\varepsilon_{v})}{{\partial \varepsilon_{v}}^{2}}+H^{2}\frac{\partial \mu }{\partial \varepsilon_{v}}-\frac{1}{2}(1+\varepsilon_{v})H^{2}\frac{\partial^{2}\mu }{{\partial \varepsilon_{v}}^{2}},\;stretch \end{array}\right.$$ (18)

Under a uniform magnetic field,

$${{\Delta E}_{v}(\varepsilon }_{v})=\left\{ \begin{array}{c} -H^{2}\frac{\partial \mu }{\partial \varepsilon_{v}}-\frac{1}{2}(1-\varepsilon_{v})H^{2}\frac{\partial^{2}\mu }{{\partial \varepsilon_{v}}^{2}},compress\\ H^{2}\frac{\partial \mu }{\partial \varepsilon_{v}}-\frac{1}{2}(1+\varepsilon_{v})H^{2}\frac{\partial^{2}\mu }{{\partial \varepsilon_{v}}^{2}},\;stretch \end{array}\right.$$ (19)

For the laminated MRE in the MR vibration isolation shock absorber, the horizontal dimension was considerably larger than the vertical dimension. The vertical strain could thus be expressed as $\varepsilon_{v}=\frac{L-l_{v}}{L}=\frac{\Delta V_{e}}{V_{t}}$. The actual particle volume fraction of the MRE under vertical deformation [40] was $\varphi^{\prime }=\frac{\varphi V_{t}}{V_{t}-\Delta V_{e}}$, where φ is the nominal particle volume fraction, *V*_*t*_ is the total MRE volume, and Δ*V*_*e*_ is the matrix deformation. Then, the actual particle volume fraction after deformation could be expressed as

$$\varphi^{\prime }=\frac{\varphi }{1-\varepsilon_{v}}b$$ (20)

where *b* is the adjustment coefficient characterizing the deformability of the nonmagnetic matrix under a vertical load. The permeability of a magnetic composite is related to the volume fraction and permeability of each component [41]:

$$\mu =\varphi^{\prime }\mu_{p}+(1-\varphi^{\prime })\varphi \mu_{e}$$ (21)

where *μ*_*p*_ and *μ*_*e*_ are the magnetic permeabilities of the particles and the nonmagnetic matrix, respectively. The relative permeability of ferromagnetic particles is generally approximately 1000 times that of nonmagnetic materials, such as rubber; thus, the aforementioned formula could be simplified as *μ* = *φ*′*μ*_*p*_. To optimise the material composition in this study, the magneto-induced tensile compression modulus was related to the particle volume fraction as follows:

$${\Delta E}_{v}=\left\{ \begin{array}{c} -2b\varphi \frac{\mu_{p}H^{2}}{{\left(1-\varepsilon_{v}\right)}^{2}},compress\\ -2b\varphi \frac{\mu_{p}H^{2}}{{\left(1+\varepsilon_{v}\right)}^{2}},\;stretch \end{array}\right.$$ (22)

**Fig 11:**
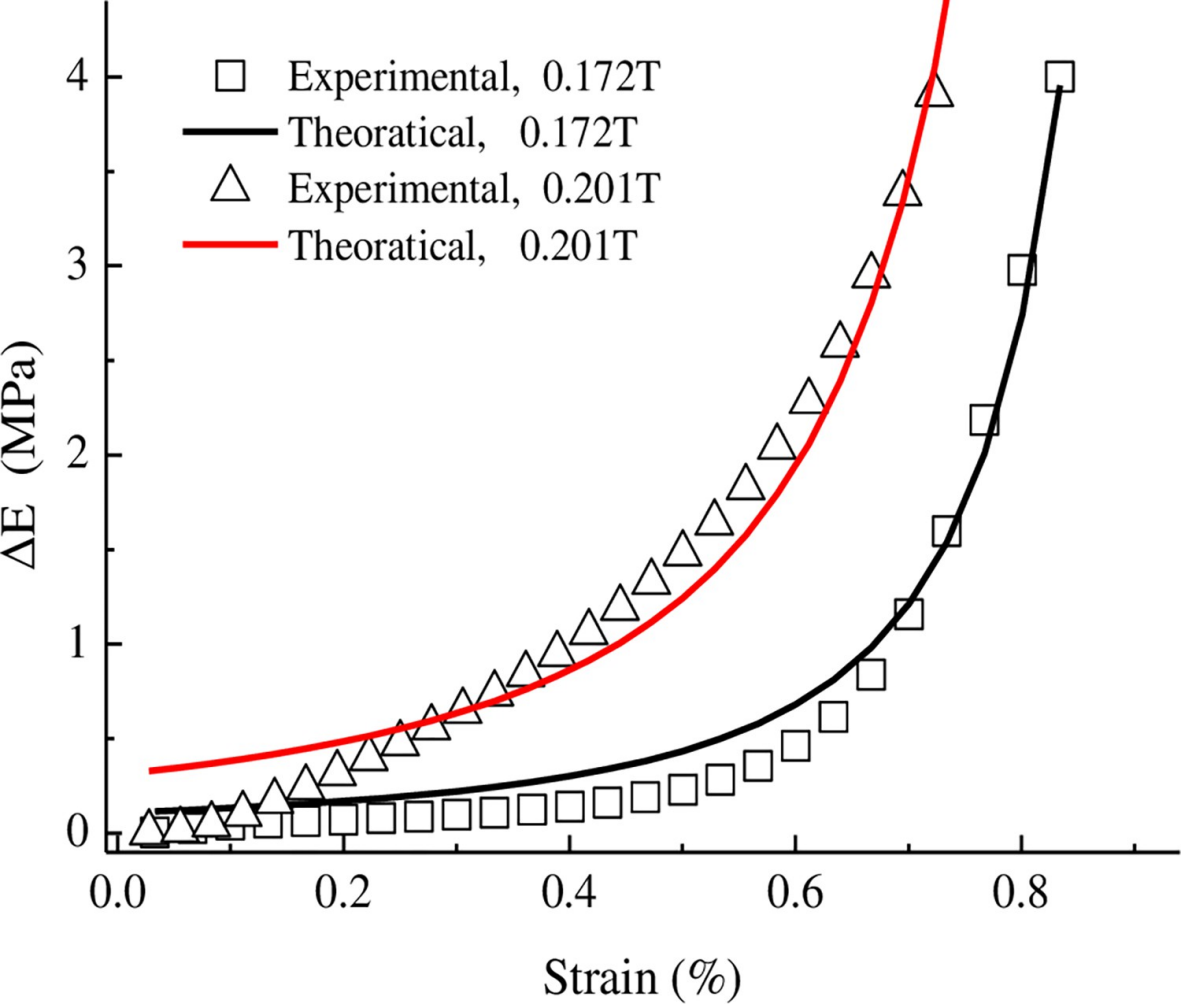
Dependence of the storage modulus on the strain under vertical compression.

The aforementioned formula shows how the ferromagnetic particle chain on hindered deformation, which manifested as the magneto-induced tensile compressive modulus increasing with the MRE elastic modulus during vertical deformation [42]. Given the working mode and force characteristics of the MRE in the MR vibration isolation support, only the magnetic compression modulus under vertical compression was fitted and analysed. The vertical strain adjustment factor *b* was 0.016 at a magnetic field strength of 0.2 T. Fig 11 shows the effect of strain on the magneto-induced modulus under vertical compression. The magneto-induced modulus of the MRE rapidly increased with the vertical compressive strain. This behaviour occurred because the distance between the individual aggregates in the MRE decreased under a small vertical compressive strain, and the compressive magneto-induced modulus increased accordingly. Beyond a vertical compressive strain of 50%, the individual aggregates were close together and squeezed each other. The distance between the ferromagnetic particles in the single aggregate decreased, such that the magneto-induced modulus increased rapidly with the strain.

## 7. Conclusions

An MR vibration isolation support was characterised in this study. The MIE mechanism of a polyether polyurethane MRE was analysed using a micromechanical model of an aggregate chain. Mathematical expressions were developed for the magnetic modulus under two working conditions, transverse shear deformation and vertical deformation. The model was validated using experimental data. The main conclusions of this study are given below.

(1) A polyurethane-rubber-based MR elastomer was prepared using rubber mixing and prestructuring. The effect of the particle volume fraction on the magneto-induced properties of the material was examined. Adding 90% carbonyl iron powder to the material changed the absolute modulus by 5 MPa and the relative modulus by 228%.

(2) Mathematical expressions were derived to describe the influence of the particle volume fraction on the magneto-induced properties of the MR elastomer under transverse shear deformation and vertical compression. These expressions were applied to the MR elastomer working unit (②, lower part) in the MRE isolation support. Under transverse shear deformation, the magneto-induced effect was greater for a particle volume fraction of 34% than for particle volume fractions of 46% and 25%. Under vertical compressive strains above 50%, the magneto-induced modulus increased rapidly with the strain.

(3) In future studies, mathematical expressions will be developed to describe the MRG magneto-induced properties of a MR plastic body working unit (①, the upper part) in an MRE isolation support under shear and extrusion working conditions. We will use mathematical expressions for the magnetic modulus under two working conditions to design and manufacture an MRG/MRE isolation device. Comprehensive performance tests will be carried out to identify essential techniques for realizing damping devices for vibration isolation.

## Supporting information

S1 Dataset Raw data supporting the results shown in Figs 5, 6, 9, and 11. (XLSX)
